# Characteristics of children with disability through infant and children’s health screening in South Korea

**DOI:** 10.1080/07853890.2025.2525401

**Published:** 2025-09-09

**Authors:** Junyoung Park, Hwa Jin Cho, Sunyong Yoo, Min-Keun Song

**Affiliations:** a Department of Intelligent Electronics and Computer Engineering, Chonnam National University, Gwangju, Republic of Korea; b Department of Pediatrics, Chonnam National University Children’s Hospital, Chonnam National University Medical School,Gwangju, Republic of Korea; c Department of Physical & Rehabilitation Medicine, Chonnam National University Medical School & Hospital, Gwangju, Republic of Korea

**Keywords:** INfants and Children’s Health screening, children with disability, developmental delay, detection of disabilities, early intervention

## Abstract

**Purpose:**

This study aimed to investigate the epidemiological data of children with disabilities obtained by the INfants and Children’s Health Screening (INCHS) program in South Korea.

**Methods:**

We conducted a retrospective case-control study by extracting data from the Korean National Health Insurance Service Database for children who were diagnosed with disabilities within 60 months of birth. Chi-square and Fisher’s exact tests were performed to compare 35,072 children born after the introduction of the INCHS program (2008–2014) with a control group born before (2002–2007). The analysis included disability registration rates by region and income, the statistical significance of timing of disability detection, and time taken to receive disability diagnosis after the INCHS program began.

**Results:**

Data on a total of 35,072 children were analyzed, revealing a significant increase (*P* < 0.001) in disability detection among the case group after 36 months compared with the control group. Although the average time to detect disabilities varied by disability type, no statistically significant difference (*P* > 0.05) was found in the proportion of hospital visits within 7 vs. 30 days between mild and severe groups. This suggests that the INCHS program can increase disability detection rates after 36 months and that there is potential for earlier disability detection.

**Conclusions:**

The INCHS program positively influenced the detection of disabilities after 36 months suggesting potential limitations in early detection. Efforts are needed to address delays in diagnosing disability and improve access to early intervention, particularly for children with mild disabilities.

## KEY MESSAGES

1. The developmental screening test is also crucial for diagnosing developmental delay and disability early.
2. The INCHS program positively influenced the detection of disabilities after 36 months of age.
3. Standardized diagnostic tools and comprehensive healthcare programs could enhance early detection and intervention, leading to improved outcomes for children with disabilities.

## 1. Introduction

Assessing growth and development is essential for growing children. Infants such as pre-term or neonatal intensive care unit babies are at risk of developmental delays and disabilities [1,2]. Particularly, since infancy and childhood are the most important developmental periods, developmental evaluation and intervention during this period may reduce the risks of future disorders and prevent secondary sequelae [3,4]. The developmental screening test is also crucial for diagnosing developmental delay and disability early. In some cases, early interventions and rehabilitation could resolve developmental delay to an almost normal level. Early detection of developmental delay needs comprehensive judgement of evaluating developmental status, medical history, and physical and neurological examination with a developmental screening test [5]. Because children differ in speed and balance of their development due to various characteristics of disabilities, further examinations are recommended. Therefore, an appropriate follow-up examination is essential. When detailed examinations are required, adequate early detection and intervention are vital for subsequent treatment.

In Japan, in accordance with Articles 12 and 13 of the Maternal and Child Health Act, municipalities conduct health examinations for infants and toddlers. Various efforts are being made to improve the health of mothers and children, including the health of infants and children, by implementing the ‘Second Healthy Maternal and Child 21’ plan. Among public health insurance programs led by the US, Medicaid and Children’s Health Insurance Program (CHIP) provide preventive health check-up programs for infants and young children. The Maternal and Child Health Bureau of the Health Resources and Services Administration (HRSA) under the Ministry of Health and Welfare developed a program (Bright Futures Program) to build a framework for improving the physical and emotional health of children [6]. In South Korea, INfants and Children’s Health Screening (INCHS) program has started nationwide since November 2007 to keep track of the growth and development of infants and children and provide proper education to caregivers, introducing a health check-up program suitable for the age of infants and children. The INCHS is conducted for the first to seventh rounds as protocols divided by the scope of a questionnaire and physical examination, physical measurements, development evaluation and consultation, health education, and oral examination. Among these, the developmental evaluation is the major screening item of INCHS, occurring during 9–12, 18–24, 30–36, 42–48, 54–60, and 66–71 months of age, all but 4–6 months, first INCHS period [7]. This program has been successfully established as one of the primary clinical services. The participation rate was 35.3% in the first year and has significantly increased to 76.7% as of 2023 [8]. Moreover, it contains protocols and questionnaires maintaining the evidence-based feature of this screening system [9]. Notably, a previous study found that 99% of children identified with suspected developmental delay during the infant screening were confirmed to have abnormal development in detailed evaluations [10].

Early identification of disability enables access to timely intervention, which significantly improves outcomes for children with disabilities and their families [11]. However, some disabilities, especially those with mild or borderline symptoms, may not be noticed until the child reaches a certain age or developmental stage. Early signs may be subtle or hidden by the child’s ability to compensate, and the disability only becomes more apparent as the child grows or faces new challenges in school or social settings [12]. Thus, there is a lack of research on the current status of children with disabilities since the INCHS program was implemented. This study aims to investigate the epidemiologic data from the National Health Insurance Service Database on children with disability since the INCHS program began and to evaluate its effectiveness as an early detection tool for the children with disability.

## 2. Methods

### 2.1. Ethical issues

The Institutional Review Board of Chonnam National University Hospital approved this study (approval no. CNUH-EXP-2020-031).

### 2.2. Definitions and severity of disability

People with disabilities can be registered in the Korean Disability Registration System based on the clinical criteria of the Welfare of Persons with Disabilities Act. The degree of disability is prescribed by the Ordinance of the Ministry of Health and Welfare. Disabilities were justified by the enforcement rule of the Act on Welfare of Persons with Disabilities [13].

Korea has a government-run grading and registration system for people with disabilities, focusing on physical and mental disabilities. Various criteria are used to grade disability severity, allowing the government to incorporate people with disabilities into the welfare system. Korea recognizes 15 types of physical and mental disabilities. Physical disabilities include brain disorders, physical disorders, visual or hearing impairments, speech disabilities; renal, cardiac, respiratory, hepatic, or facial disorders; intestinal or urinary fistula, and epilepsy. Intellectual disabilities and autistic or psychological disorders are recognized as mental disabilities [14].

In the Korean Disability Registration and Grading System, criteria for judging the degree of disability are set according to disability characteristics. Diagnosis is made when the disorder is alleviated after administering treatment specific to the supposed condition. The standard period is 6 months after disease onset, injury, or continuous treatment for >6 months after surgery. However, cases when the fixation of a disability is apparent, such as amputation of a body part or spinal fixation, represent exceptions. The Korean Disability Registration and Grading System classified disability into severity grades 1–6 after a medical examination based on disability type to provide various welfare systems and support for people with disabilities in 1998. However, in July 2019, the disability grading system was abolished due to several problems, such as the inability to adequately provide services tailored to individuals with disabilities. The grading of disability severity was changed to include only minimal divisions, such as grades 1–3 for those with severe disabilities and grades 4–6 for those with mild disabilities [15]. Therefore, we classified the subjects in this study based on their disability severity, i.e. mild or severe. Mild disability included people who performed daily tasks alone, although they partly needed personal assistance or assistive devices. Severe disability was defined as high dependence on personal assistance or assistive devices to perform daily tasks [14]. In Korea, the government provides different welfare services according to the severity of the disability.

### 2.3. Research design

This study employed a retrospective case-control design to compare children with disabilities born before the nationwide introduction of the INCHS program (2002–2007) with those born after its implementation (2008–2014), analysing differences between the pre- and post-program periods. The data for children with disability in the Korean disability registration system were extracted from the Korean National Health Insurance Service Database. New cut-off points were determined after analysing the average, median, standard deviation, maximum, minimum, and score distribution for subdomains of each age group. Since this test was designed to distinguish infants and children with developmental delays, infants with normal development mostly had high test scores. For the case group, children with disability diagnosed before 60 months and born between 2008 and 2014 who participated in the INCHS program were selected. The control group comprised children with disabilities diagnosed before 60 months and born between 2002 and 2007 who did not participate in the INCHS program.

### 2.4. Aims and objectives

We analyzed the general characteristics of the study population, including gender, age, region and insurance premium brackets. Then, we investigated disability registration rates by region and income level, categorizing the income basket into high (≥16th), middle (8th–15th), and low (≤7th) income for comparison. We also examined the average time to detect disabilities, focusing on the detection rates across various disability types. Finally, hospital visit patterns after the introduction of the INCHS program were assessed, comparing the time to visit a hospital by disability type and severity.

### 2.5. Data analysis

Chi-square tests were conducted to determine whether the distributions of general characteristic variables (gender, age, region and insurance premium brackets) differed significantly between the case and control groups. Descriptive statistics (i.e. frequencies and percentages) were primarily employed for regional and income-based comparisons. Both the time taken to detect disabilities and the post-INCHS hospital visit patterns were analyzed using proportions stratified by disability type and severity. Significance tests were performed using either Pearson’s Chi-squared test or Fisher’s exact test, depending on the sample size and expected frequencies within each contingency table. Specifically, the Chi-squared test was applied when analysing (1) income-based disability registration rates and (2) the time taken to detect disabilities, as these comparisons involved sufficiently large sample sizes and met the usual assumptions for Chi-squared (i.e. expected cell counts ≥5). Conversely, Fisher’s exact test was used to analyse (3) post-INCHS hospital visit patterns by disability type and severity, as some subgroups had smaller counts, making Fisher’s exact test more suitable for cases with small sample sizes or low expected frequencies. Statistical analyses were conducted using Python 3.7 and the packages NumPy (version 1.21.6), Pandas (version 1.3.5), SciPy (version 1.7.3) and Statsmodels (version 0.13.5).

## 3. Results

### 3.1. General characteristics

**Figure 1.**
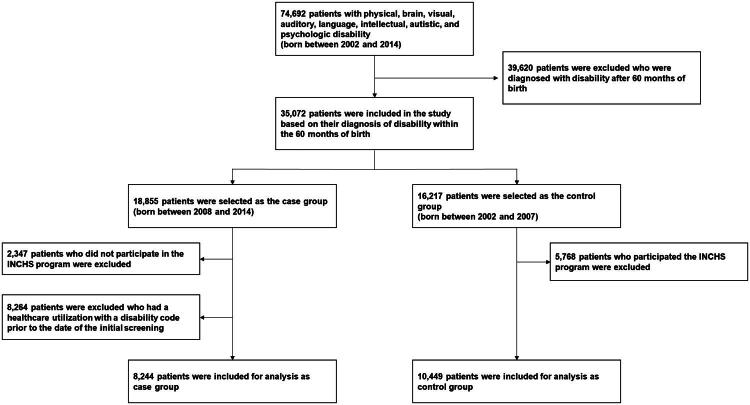
Flow diagram of study participants.

We selected 74,692 patients with physical, brain, visual, auditory, language, intellectual, autistic, and psychologic disabilities from a population of 6,087,782 children born between 2002 and 2014. Among these, the study focused on 35,072 patients diagnosed with a disability within the 60 months of birth, specifically targeting early-detected cases through the INCHS program, which conducts health screenings and developmental monitoring during the children’s first five years. The case and control groups were categorized based on birth year, with those born before 2008 assigned to the control group and those born from 2008 onward to the case group. Out of 35,072 patients, 16,217 were initially assigned to the control group, while the remaining 18,855 were assigned to the case group. However, 8264 patients from the case group were excluded because they had utilized healthcare services with a disability code prior to the date of the initial INCHS screening. Additionally, 5768 patients who participated in the INCHS program were excluded from the control group, and 2347 patients who did not participate in the INCHS program were excluded from the case group. These exclusions were required be­cause INCHS participation status is the key exposure variable. By definition, the control group represents children who did not participate in the INCHS program, whereas the case group represents children who did. Finally, we generated the control group of 10,449 patients and the case group of 8244 patients. Figure 1 shows the flowchart of study participants and data preparation.

The case group included 5800 males and 2444 females. The control group comprised 6458 males and 3991 females. In the case group, participants residing in Gyeonggi-do were the most numerous, accounting for 2078 individuals, followed by Seoul with 1585 participants. In contrast, Seoul had the highest number of participants (2907) in the control group, followed by Gyeonggi-do (2190). There were no participants from Sejong. Excluding these two regions, the number of participants from all other areas was <1000. From the perspective of insurance premium brackets, the case group had the highest number of participants in the 14^th^ bracket, while the control group had the most participants in the 10^th^ bracket. In the Korean National Health Insurance system, insurance premiums are divided into 20 brackets based on income rank. Higher insurance premium brackets indicate higher household income. Chi-square tests revealed statistically significant differences in gender, age group, region, and insurance premium bracket distributions between the case and control groups (*P* < 0.001). Table 1 characterizes each group.

**Table 1.** Basic characteristics of participants in each group.

		Case group (*N* = 8244)	Control group (*N* = 10,449)	*p* Value
Gender (%)				<0.001
	Male	5800 (70.4)	6458 (61.8)	
	Female	2444 (29.6)	3991 (38.2)	
Age (%)				<0.001
	0–3 Months	5 (0.1)	116 (1.1)	
	4–6 Months	22 (0.3)	295 (2.8)	
	7–9 Months	62 (0.8)	416 (4.0)	
	10–12 Months	69 (0.8)	585 (5.6)	
	13–24 Months	931 (11.3)	2632 (25.2)	
	25–36 Months	1220 (14.8)	2163 (20.7)	
	37–48 Months	2343 (28.4)	2222 (21.3)	
	49–60 Months	3592 (43.6)	2020 (19.3)	
Region (%)				<0.001
	Null	102 (1.2)	431 (4.1)	
	Seoul	1585 (19.2)	2907 (27.8)	
	Busan	497 (6.0)	508 (4.9)	
	Daegu	304 (3.7)	412 (3.9)	
	Incheon	525 (6.4)	503 (4.8)	
	Gwangju	175 (2.1)	235 (2.2)	
	Daejeon	257 (3.1)	289 (2.8)	
	Ulsan	178 (2.2)	190 (1.8)	
	Sejong	7 (0.1)	0 (0.0)	
	Gyeonggi-do	2078 (25.2)	2190 (21)	
	Gangwon-do	245 (3.0)	303 (2.9)	
	Chungcheongbuk-do	244 (3.0)	296 (2.8)	
	Chungcheongnam-do	389 (4.7)	383 (3.7)	
	Jeollabuk-do	256 (3.1)	316 (3.0)	
	Jeollanam-do	254 (3.1)	297 (2.8)	
	Gyeongsangbuk-do	418 (5.1)	487 (4.7)	
	Gyeongsangnam-do	597 (7.2)	582 (5.6)	
	Jeju Island	133 (1.6)	120 (1.1)	
Insurance premium bracket (%)				<0.001
	Null	284 (3.4)	243 (2.3)	
	0	186 (2.3)	318 (3.0)	
	1	248 (3.0)	319 (3.1)	
	2	175 (2.1)	211 (2.0)	
	3	190 (2.3)	237 (2.3)	
	4	186 (2.3)	256 (2.4)	
	5	253 (3.1)	315 (3.0)	
	6	282 (3.4)	459 (4.4)	
	7	331 (4.0)	533 (5.1)	
	8	372 (4.5)	577 (5.5)	
	9	430 (5.2)	568 (5.4)	
	10	506 (6.1)	781 (7.5)	
	11	566 (6.9)	725 (6.9)	
	12	545 (6.6)	772 (7.4)	
	13	591 (7.2)	759 (7.3)	
	14	606 (7.4)	717 (6.9)	
	15	601 (7.3)	697 (6.7)	
	16	534 (6.5)	577 (5.5)	
	17	491 (6.0)	469 (4.5)	
	18	370 (4.5)	392 (3.8)	
	19	292 (3.5)	282 (2.7)	
	20	205 (2.5)	242 (2.3)	

### 3.2. Disability registration rates by region and income

**Figure 2.**
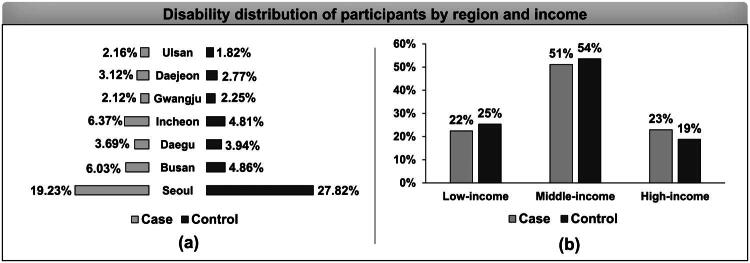
Comparison of disability distribution for each case between case and control groups. (a) Comparison of region distribution between the groups. (b) Comparison of income distribution between the groups.

We analyzed the disability registration rates within 60 months of age for 17 regions in Korea (Supplementary Figure 1). Among these regions, we conducted a more detailed analysis of 7 major metropolitan cities, in­­cluding the capital. The registration rate of disability tended to decrease mainly in the capital (Figure 2(a)). Specifically, Seoul, the capital, exhibited the largest decrease of 8.59%, while Daegu and Gwangju had smaller decreases of 0.25% and 0.13%, respectively. Among the remaining regions, Incheon had the highest increase in the registration rate of 1.56%, while Ulsan had the lowest increase rate of 0.34%. Comparing the two groups by insurance premium bracket, the difference rate was <1.5% in all brackets (Supplementary Figure 2). We compared the disability enrolment rate across income-based three groups. The rate increased by about 4% for the high-income group, while decreasing by 3% in both low- and middle-income groups (Figure 2(b), Supplementary Table 1). Additionally, Chi-square tests revealed a significant difference (*P* < 0.001) in the overall distribution of disability registration rates between the case and control groups.

### 3.3. Average time spent detecting disabilities by disability type after the INCHS program started

**Figure 3.**
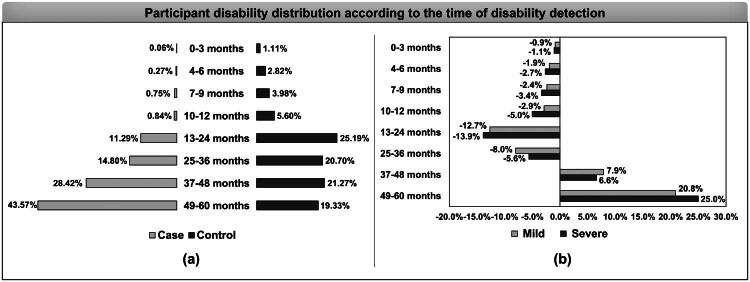
Comparative analysis of disability distribution in each case for case and control groups. Since participants in the groups were different, all statistical data were expressed as percentages. The results are presented based on these percentages. (a) Comparison of the distribution of the time of disability detection after birth between the two groups. (b) Comparison of the distribution of the time of disability detection after birth by disability grade between the two groups.

There was a clear difference between the two groups in the timing of disability detection for infants and children after birth. In the control group, participants aged 0–36 months accounted for 59.4%, but this decreased to about 28% in the case group. Among them, the number of participants aged 13–24 months showed the largest decrease of 13.9%. In contrast, the number of participants aged 37–48 months increased by 7.15% in the case group compared to the control group, and the number of participants aged 49–60 months increased by 24.2% (Figure 3(a), Table 2). Thus, the INCHS program may have a positive effect on the detection of disabilities for >36 months of age. Severe brain disabilities were most frequently identified between 13 and 24 months in the case group, with 511 children recorded in that interval (Table 3). Severe autism represented the opposite extreme, and 1438 children were recorded between 49 and 60 months in the case group, whereas 537 children were recorded in the control group (Tables 3 and 4). Physical disabilities followed a similar pattern, because the control group included 25 children with severe disability within the first three months of life, whereas none were recorded in the case group during the same period (Tables 2 and 4). Across several disability categories, detections after 37 months were consistently more common in the case group, while the control group dominated the earlier age intervals (Table 2). These observations collectively suggest a systematic right shift in the age of disability recognition following the nationwide introduction of INCHS program.

**Table 2.** Distribution of disability registrations by age at diagnosis in case and control groups.

		Case group (*N* = 8244)	Control group (*N* = 10,449)	*p* Value
Months after birth				<0.001
	0–3 Months	5	116	
	4–6 Months	22	295	
	7–9 Months	62	416	
	10–12 Months	69	585	
	13–24 Months	931	2632	
	25–36 Months	1220	2163	
	37–48 Months	2343	2222	
	49–60 Months	3592	2020	

**Table 3.** Age at diagnosis by disability type and severity in the case group.

Type	Grade	0–3 Months	4–6 Months	7–9 Months	10–12 Months	13–24 Months	25–36 Months	37–48 Months	49–60 Months	Total
Physical (%)	Severe	–	1 (0.01)	3 (0.04)	2 (0.02)	18 (0.22)	15 (0.18)	11 (0.13)	10 (0.12)	60 (0.73)
	Mild	–	1 (0.01)	1 (0.01)	5 (0.06)	17 (0.21)	20 (0.24)	20 (0.24)	24 (0.29)	88 (1.07)
Brain (%)	Severe	–	2 (0.02)	8 (0.10)	16 (0.19)	511 (6.20)	301 (3.65)	118 (1.43)	74 (0.90)	1030 (12.49)
	Mild	–	–	1 (0.01)	3 (0.04)	93 (1.13)	111 (1.35)	70 (0.85)	55 (0.67)	332 (4.04)
Visual (%)	Severe	–	–	3 (0.04)	2 (0.02)	7 (0.08)	14 (0.17)	4 (0.05)	12 (0.15)	42 (0.51)
	Mild	–	3 (0.04)	3 (0.04)	1 (0.01)	27 (0.33)	24 (0.29)	23 (0.28)	13 (0.16)	94 (1.14)
Auditory (%)	Severe	5 (0.06)	10 (0.12)	34 (0.41)	31 (0.38)	156 (1.89)	85 (1.03)	57 (0.69)	27 (0.33)	405 (4.91)
	Mild	–	3 (0.04)	7 (0.08)	2 (0.02)	12 (0.15)	22 (0.27)	36 (0.44)	30 (0.36)	112 (1.36)
Language (%)	Severe	–	–	1 (0.01)	1 (0.01)	17 (0.21)	34 (0.41)	149 (1.81)	152 (1.84)	354 (4.29)
	Mild	–	1 (0.01)	–	1 (0.01)	15 (0.18)	84 (1.02)	407 (4.94)	595 (7.22)	1103 (13.38)
Intellectual (%)	Severe	–	1 (0.01)	1 (0.01)	5 (0.06)	50 (0.61)	307 (3.72)	675 (8.19)	1162 (14.10)	2201 (26.70)
Autistic (%)	Severe	–	–	–	–	8 (0.10)	203 (2.46)	773 (9.38)	1438 (17.44)	2422 (29.38)

**Table 4.** Age at diagnosis by disability type and severity in the control group.

Type	Grade	0–3 Months	4–6 Months	7–9 Months	10–12 Months	13–24 Months	25–36 Months	37–48 Months	49–60 Months	Total
Physical (%)	Severe	25 (0.24)	54 (0.52)	43 (0.41)	44 (0.42)	116 (1.11)	109 (1.04)	59 (0.56)	55 (0.53)	105 (4.83)
	Mild	8 (0.08)	10 (0.10)	10 (0.10)	17 (0.16)	60 (0.57)	76 (0.73)	96 (0.92)	55 (0.53)	332 (3.18)
Brain (%)	Severe	24 (0.23)	89 (0.85)	180 (1.72)	312 (2.99)	1444 (13.82)	685 (6.56)	290 (2.78)	120 (1.15)	3144 (30.09)
	Mild	–	1 (0.01)	7 (0.07)	15 (0.14)	163 (1.56)	136 (1.30)	80 (0.77)	55 (0.53)	457 (4.37)
Visual (%)	Severe	4 (0.04)	10 (0.10)	13 (0.12)	13 (0.12)	58 (0.56)	49 (0.47)	32 (0.31)	25 (0.24)	204 (1.95)
	Mild	3 (0.03)	13 (0.12)	15 (0.14)	11 (0.11)	55 (0.53)	51 (0.49)	68 (0.65)	55 (0.53)	271 (2.59)
Auditory (%)	Severe	8 (0.08)	29 (0.28)	47 (0.45)	39 (0.37)	224 (2.14)	180 (1.72)	79 (0.76)	57 (0.55)	663 (6.35)
	Mild	1 (0.01)	9 (0.09)	8 (0.08)	5 (0.05)	22 (0.21)	22 (0.21)	27 (0.26)	33 (0.32)	127 (1.22)
Language (%)	Severe	–	1 (0.01)	2 (0.02)	2 (0.02)	16 (0.15)	59 (0.56)	85 (0.81)	58 (0.56)	223 (2.16)
	Mild	–	–	2 (0.02)	1 (0.01)	4 (0.04)	30 (0.29)	62 (0.59)	85 (0.81)	184 (1.76)
Intellectual (%)	Severe	41 (0.39)	74 (0.71)	85 (0.81)	117 (1.12)	424 (4.06)	542 (5.19)	820 (7.85)	885 (8.47)	2988 (28.60)
Autistic (%)	Severe	2 (0.02)	5 (0.05)	4 (0.04)	9 (0.09)	46 (0.44)	224 (2.12)	524 (5.01)	537 (5.14)	1351 (12.91)

The time of disability detection was further analyzed by disability type, including physical disability, brain disability, visual disability, hearing disability, language disability, intellectual disability and autism spectrum disability. For the same criteria, a detailed analysis was conducted on the increase and decrease in the time of disability detection by subtracting the participant proportion of the control group from that of the case group. Comparing the two groups, the time of disability detection for participants aged <12 months old decreased across all disability types. Participants aged 13–24 months showed a slight increase of 0.2% for language disability, with a decrease in all other cases. For language disability, there was an increase in the disability detection rate after the age of 13 months. Participants with autistic spectrum disability aged 49–60 months showed the highest increase of 12.3%, while those with auditory disabilities aged 37–48 months had the lowest increase of 0.11%.

Each group was divided by disability grade for comparative analysis (Supplementary Table 2). Following the previous method, the change was specifically analyzed by subtracting the percentage of participants in the control group from that in the case group for the same grade. Figure 3(b) shows the specific changes, where a negative number indicates a decrease compared to the control group. Participants aged <37 months exhibited decreases across all grades, with the most significant decline (13.9%) in participants aged 13–24 months with severe disability. In contrast, participants aged >37 months showed an increase in all grades, with those aged 49–60 months with severe disability showing the largest increase of 25%. We conducted Chi-square tests on detection times and disability grade, revealing a statistically significant difference (*P* < 0.001) between the case and control groups.

### 3.4. The time taken to receive the first diagnosis of a disability after the INCHS program started

**Figure 4.**
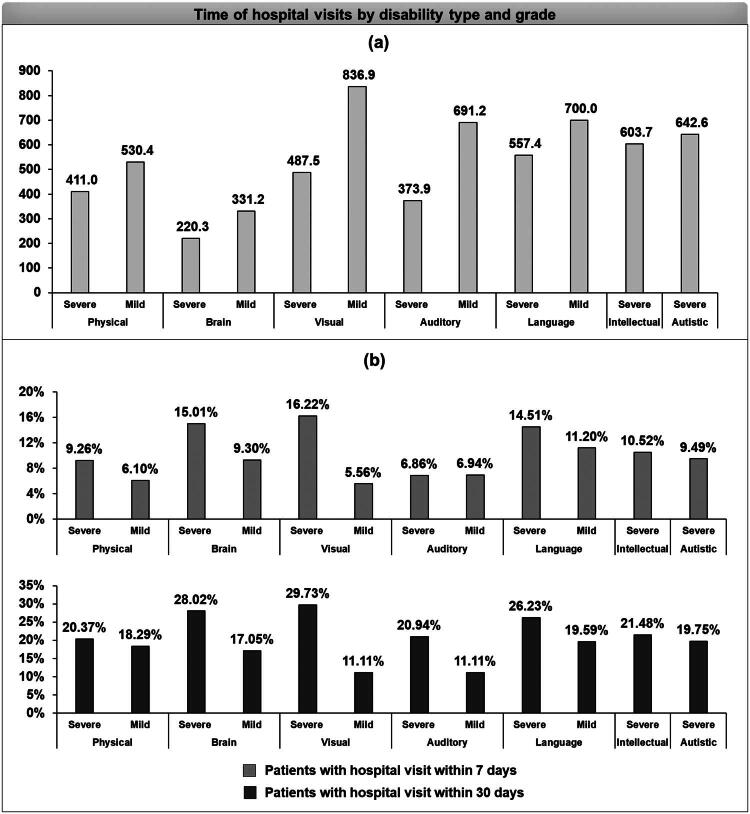
Comparison of hospital visit times by disability type and grade in the case group. (a) Comparison of average hospital visit times for each type and grade. (b) The proportion of hospital visits by time period for participants. The proportions of hospital visitors within 7 and 30 days were indicated.

We checked the time taken to receive the initial diagnosis of disability following the INCHS program examination (Figure 4). Children with mild visual disabilities experienced the longest time before being diagnosed, approximately 836.9 days after the INCHS program started. Moreover, there was a noticeable difference of about 349.4 days in the time spent on diagnosis after the INCHS program began between severe and mild visual disabilities. This represented the longest duration among the case group patients, with a difference of approximately 136.9 days compared to language disability patients who had the second-longest duration. In the case of brain disabilities, both severe and mild grades showed the shortest average hospital visit duration, with severe cases waiting for about 220.3 days and mild cases waiting for 331.2 days after the initial examination. Overall, all disability types except for brain disability resulted in hospital visits occurring after one year. Furthermore, the difference between severe and mild patients was <150 days in other disability types excluding visual disability and auditory disability (Figure 4(a)).

We analyzed the proportion of hospital visits occurring within 7 and 30 days after the initial INCHS examination to evaluate the program’s impact on the promptness of care. This was performed by categorizing the case group patients by disability type and level, finding the number of visits within 7 and 30 days, and deriving the percentages. We used percentages rather than counts to make the analysis more readable and easier to compare. From the original case group, 914 individuals were excluded due to the absence of disability diagnostic codes, resulting in a total of 7330 patients for this analysis. For visual disability, the number of severe patients was the highest at 16.22% and 29.73%, respectively (Figure 4(b)). Thus, among the 37 severe patients, 6 visited the hospital within 7 days, and 11 visited the hospital within 30 days. On the contrary, the results for mild patients were 5.56% and 11.11%, respectively, showing the lowest rates across all disability types and grades. Therefore, visual disability is the type with the largest difference between severe and mild patients. The number of visitors within 7 and 30 days was generally higher in the severe groups across most disability types (Supplementary Table 3).

Next, we conducted Fisher’s exact test to determine whether the proportion of hospital visits within 7 days differs significantly from the proportion of visits within 30 days for each disability type and severity level (Table 5). The result indicated that none of the disability types showed a *p* value below 0.05, suggesting that the difference in visit rates within 7 and 30 days between severe and mild groups was not statistically significant. For instance, physical disability had a *p* value of 0.7225, while auditory (*P* = 0.3204) and language (*P* = 0.9145) also showed no significant differences. Similarly, the *p* values for brain (*P* = 1.0) and visual (*P* = 1.0) disability groups exceeded 0.05. This finding suggests that the INCHS program may have a latent capacity to facilitate earlier detection and hospital visits, not only for severe disabilities but also for mild disabilities.

**Table 5.** Difference in the timing of hospital visits after the INCHS according to disability type and severity.

Disability type		Visitors within 7 days	Visitors within 30 days	*p* Value
Physical	Severe	5	11	0.7225
	Mild	5	15	
Brain	Severe	112	209	1
	Mild	24	44	
Visual	Severe	6	11	1
	Mild	5	10	
Auditory	Severe	19	58	0.3204
	Mild	5	8	
Language	Severe	47	85	0.9145
	Mild	116	203	

## 4. Discussion

Based on the extracted data, we analyzed the registration period for each disability type, grade, and type and grade after launching the INCHS program. The study showed the impact of the Infants and Children’s Health Screening program on childhood disability detection rates in South Korea. Analysis of data from over 10,000 children revealed a significant increase in the detection of developmental delays and disabilities following the implementation of the screening program. Additionally, differences in gender distribution, region of residence, and insurance premium brackets were identified between the two groups.

The comparison by income showed varying enrolment rates: an increase of about 4% for the high-income group and a decrease of 3% for low- and middle-income groups. Many countries, particularly low- and low- to middle-income countries, do not have the resources to develop or conduct screening based on context-specific measures [16]. Those countries may be associated with an increased incidence of very-low-birth-weight infants at a higher risk of neurodevelopmental delay [17]. Since loss to follow-up frequently occurs mainly owing to high examination costs and transfers to other institutes, setting up a standardized diagnostic tool, protocol, and official healthcare program can be effective in subsequent diagnosis and treatment, as well as tracking the results [18]. This result may impact the delay in evaluating and treating children with disabilities.

One of the notable results is the increase in disability detection rates after 36 months of age among children born after the implementation of the INCHS program. Thus, the program might have raised awareness among parents and healthcare professionals, leading to more timely identification of disabilities. However, this increase in disability detection was primarily observed after 36 months, indicating potential limitations in early detection efforts, which is concerning. First, the reason why the increase in disability detection rates showed after the age of 36 months is that detection of mild disabilities, especially developmental delay, may be difficult in children aged <60 months [19]. Barrier reported a lack of reimbursement for the 30-month visit [20]. Second, qualified tools for evaluating developmental delays or disabilities were lacking before the INCHS program began. Screening should be performed with qualified tools to minimize under-detection and over-referrals [21]. After launching the INCHS, children with mild developmental delays or disabilities could be detected more frequently by using validated screening tools.

Furthermore, the study revealed that the time spent on diagnosis varied across disability types and grades, with brain disabilities showing the shortest average hospital visit durations. The proportion of hospital visits within 7 and 30 days varied by disability type and severity. However, it is still considered that institutional help is needed for the long time it takes to visit a medical institution after the infant health check-up and the rate of visits within 30 days is low. If early rehabilitation is performed for children with disabilities, the treatment effect is substantial. Hence, systematizing the visit to the department of rehabilitation medicine for a precise diagnosis after the infant health check-up is essential. However, national support is needed because the department of rehabilitation medicine is responsible for both diagnosis and treatment, the clinical pattern of children continues to change with growth and development even if it is not a progressive disease, direct communication with the patient is often difficult, and paediatric rehabilitation is considered one of the areas that require the most expertise and is difficult to access.

This suggests that while the program might increase overall awareness and access to healthcare services, there are still barriers preventing some children from receiving timely medical attention. Addressing these barriers, such as improving accessibility to healthcare facilities and promoting community outreach programs, is essential for ensuring equitable access to early intervention services. The results also underscore the importance of comprehensive healthcare programs going beyond early screening to provide ongoing support for children with disabilities and their families. Early detection is only the first step, with effective intervention and support services being crucial for optimizing developmental outcomes and enhancing the quality of life for children with disabilities. This includes access to specialized healthcare professionals, therapeutic interventions, educational resources, and social support networks tailored to meet the diverse needs of children with different disability types and severities. However, the existing medical system does not adequately improve the health and quality of life of children with disability [22]. Korean legislation on medical welfare for people with disabilities promotes the rights of people with disabilities and encourages improvements in medical amenities, accessibility, awareness, and provision of financial assistance when people with disabilities require clinical attention [23]. The required medical services differ according to the developmental stage of children with disabilities. A previous study reported that children with disabilities had a higher prevalence of congenital defects and decline in their health condition and a lower prevalence of infectious diseases than children without disabilities; therefore, they use more medical services, have higher hospitalization rates, more comorbidities, and higher medical expenses [24,25]. Furthermore, caregivers may not continue rehabilitative management for children with disabilities because of the related family burden [26]. Therefore, an integrated delivery system in connection with various resources in the community is required, such as life-long health care and high-qualified rehabilitation medical services for each life cycle of children with disabilities, special education services, rehabilitation sports services, and family support services, which are important factors for the health and life of children with disabilities. Thus, efforts are urgently needed to register disability through infant screening and establish an optimal rehabilitation and treatment delivery system accordingly. The importance of early rehabilitation management based on brain plasticity cannot be overemphasized, as well as the provision of an appropriate treatment environment. Moreover, when a child with a developmental delay or disability is registered in the national database system, children with disabilities could receive total care management such as medical, educational, and vocational rehabilitation services according to appropriate and effective guidelines throughout the lifecycle.

This study has some limitations. Firstly, the sample was restricted to individuals in South Korea, potentially limiting the generalizability of the findings to other populations. Secondly, the analysis did not fully account for the influence of key demographic factors, such as family characteristics and access to medical services. Thirdly, the potential impact of comorbid disabilities was not considered. Nevertheless, the findings of this analysis, which subdivide children with disabilities, can serve as valuable reference material for the standardization of welfare services, enabling the provision of more accurate and effective support.

## 5. Conclusions

After the INCHS program started, previously undiagnosed children with disabilities could receive benefits from registering their disabilities, especially noncapital region patients. Although the INCHS program showed a statistically significant increase in disability detection after 36 months (*P* < 0.001), there was no significant improvement (*P* = 0.23) in the detection time for younger children (<36 months). Several factors may explain this limited improvement, including (i) subtle or nonspecific clinical signs in early infancy, (ii) lower parental awareness of early-stage symptoms, and (iii) delays in accessing specialist for confirmative diagnosis, especially as families living far from the metropolitan areas. To address these issues, the program could incorporate age-specific follow-up visits, and strengthen primary-care referral pathways so that children who screen positive reach specialists more quickly. Future research should also stratify children with common comorbidities such as cerebral palsy with epilepsy or autism with intellectual disability. This approach would clarify whether comorbidities affects detection timing and help identify additional barriers to early, comprehensive rehabilitation.

## Supplementary Material

Supplemental Material

## Data Availability

This study’s supporting data are available through the Health Resources and Services Administration of the Ministry of Health and Welfare, Korea; however, restrictions apply. These data were used under licence for the current study and are not publicly available. Data are available from the corresponding author upon reasonable request.
