# Supplementary material for: Characteristics of children with disability through infant and children’s health screening in South Korea
Source: Ann Med. 2025 Sep 9;57(1):2525401. doi: 10.1080/07853890.2025.2525401 (PMC12422035; doi:10.1080/07853890.2025.2525401)
Supplement: Supplemental Material [file IANN_A_2525401_SM4213.docx]

**Supplementary**


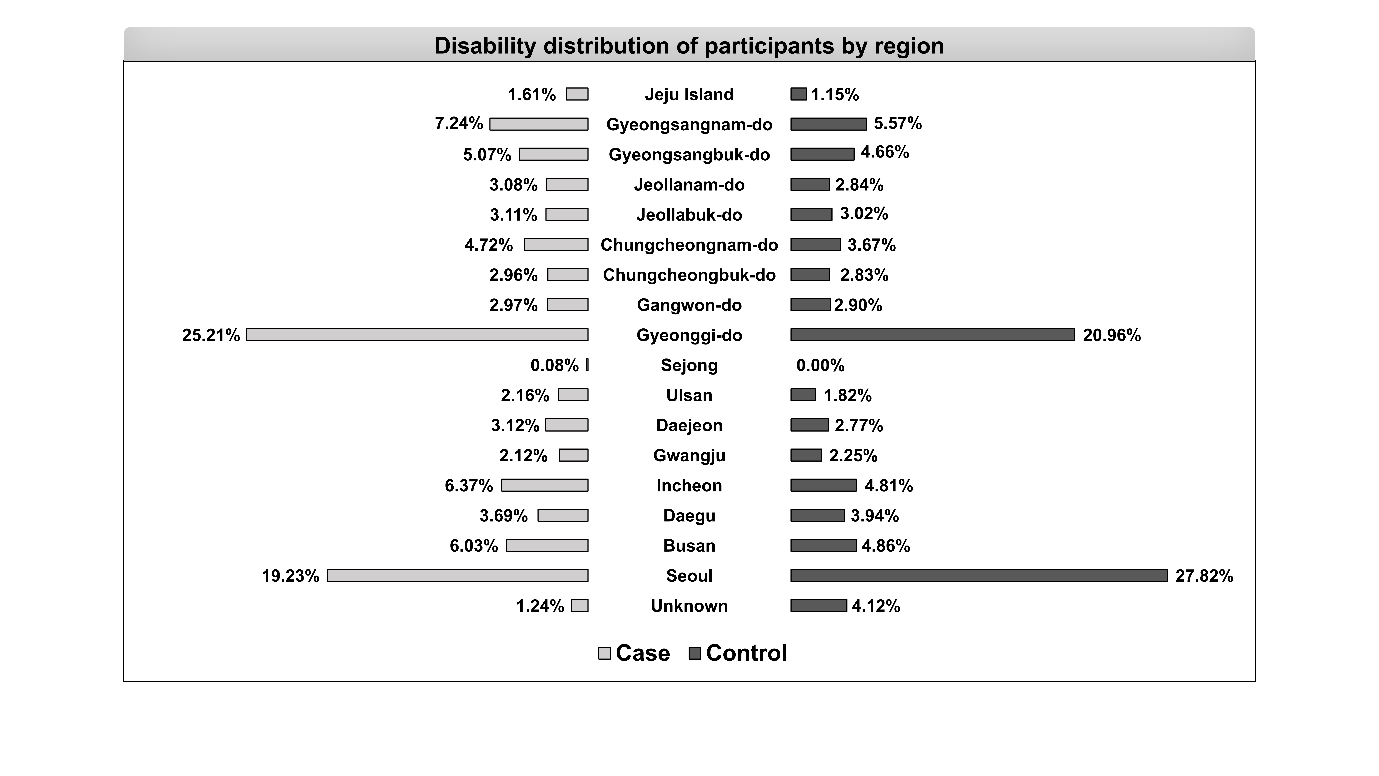


**Supplementary Figure 1**. Number of study participants by region for case and control groups


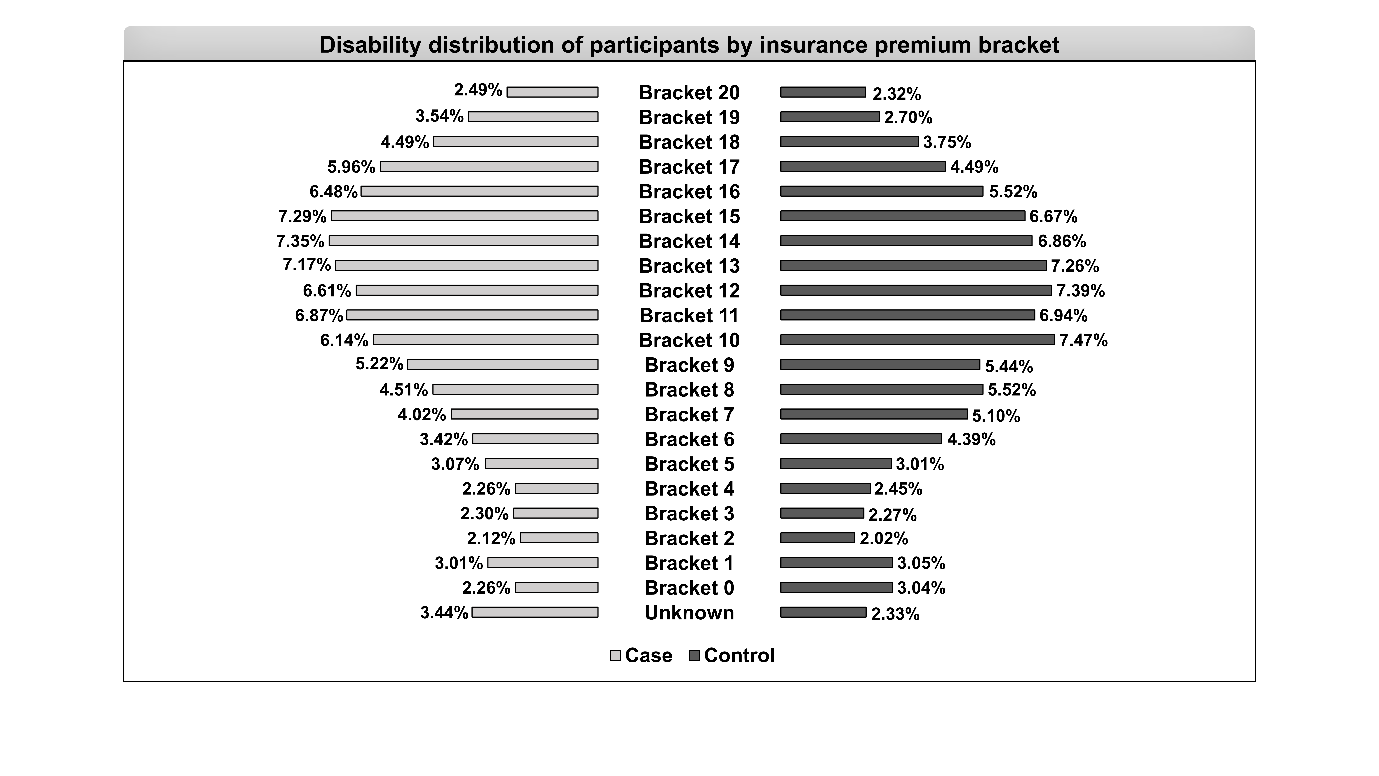


**Supplementary Figure 2**. Number of study participants by insurance premium bracket for case and control groups.

**Supplementary Table 1**. Number of participants per income level in the case and control groups.

|  |  | **Case group**  **(N=8,244)** | **Control group**  **(N=10,449)** | ***P* value** |
| --- | --- | --- | --- | --- |
| Income |  |  |  | < 0.001 |
|  | Low | 1,851 | 2,648 |  |
|  | Middle | 4,217 | 5,596 |  |
|  | High | 1,892 | 1,962 |  |

|  |  | **Case group**  **(N=8,244)** | | **Control group**  **(N=10,449)** | |  |
| --- | --- | --- | --- | --- | --- | --- |
| Age |  | **Severe** | **Mild** | **Severe** | **Mild** | ***P* value** |
|  |  |  |  |  |  | < 0.001 |
|  | 0–3 months | **5** | **0** | **104** | **12** |  |
|  | 4–6 months | **14** | **8** | **262** | **33** |  |
|  | 7–9 months | **50** | **12** | **374** | **42** |  |
|  | 10–12 months | **57** | **12** | **536** | **49** |  |
|  | 13–24 months | **767** | **164** | **2,328** | **304** |  |
|  | 25–36 months | **959** | **261** | **1,846** | **317** |  |
|  | 37–48 months | **1,787** | **556** | **1,889** | **333** |  |
|  | 49–60 months | **2,875** | **717** | **1,737** | **283** |  |

**Supplementary Table 2**. Distribution of disability detection time by severity in the case and control groups.

**Supplementary Table 3.** Number of hospital visits by the cases according to disability type and severity.

| **Disability type** | | **Total** | **Visitors**  **within 7 days** | **Visitors**  **within 30 days** |
| --- | --- | --- | --- | --- |
| Physical | Severe | 54 | 5 | 11 |
|  | Mild | 82 | 5 | 15 |
| Brain | Severe | 746 | 112 | 209 |
|  | Mild | 258 | 24 | 44 |
| Visual | Severe | 37 | 6 | 11 |
|  | Mild | 90 | 5 | 10 |
| Auditory | Severe | 277 | 19 | 58 |
|  | Mild | 72 | 5 | 8 |
| Language | Severe | 324 | 47 | 85 |
|  | Mild | 1,036 | 116 | 203 |
| Intellectual | Severe | 2,025 | 213 | 435 |
| Autistic | Severe | 2,329 | 221 | 460 |
